# Trafficking to the Cell Surface of Amino Acid Transporter SLC6A14 Upregulated in Cancer Is Controlled by Phosphorylation of SEC24C Protein by AKT Kinase

**DOI:** 10.3390/cells10071800

**Published:** 2021-07-16

**Authors:** Vasylyna Kovalchuk, Katarzyna A. Nałęcz

**Affiliations:** Nencki Institute of Experimental Biology, Polish Academy of Sciences, 3 Pasteur Street, PL-02-093 Warsaw, Poland; v.kovalchuk@nencki.edu.pl

**Keywords:** amino acid transporter, SLC6A14, AKT kinase, SEC24C, breast cancer

## Abstract

Cancer cells need a constant supply of nutrients. SLC6A14, an amino acid transporter B^0,+^ (ATB^0,+^) that is upregulated in many cancers, transports all but acidic amino acids. In its exit from the endoplasmic reticulum (ER), it is recognized by the SEC24C subunit of coatomer II (COPII) for further vesicular trafficking to the plasma membrane. SEC24C has previously been shown to be phosphorylated by protein kinase B/AKT, which is hyper-activated in cancer; therefore, we analyzed the influence of AKT on SLC6A14 trafficking to the cell surface. Studies on overexpressed and endogenous transporters in the breast cancer cell line MCF-7 showed that AKT inhibition with MK-2206 correlated with a transient increase of the transporter in the plasma membrane, not resulting from the inhibition of ER-associated protein degradation. Two-dimensional electrophoresis demonstrated the decreased phosphorylation of SLC6A14 and SEC24C upon AKT inhibition. A proximity ligation assay confirmed this conclusion: AKT inhibition is correlated with decreased SLC6A14 phosphothreonine and SEC24C phosphoserine. Augmented levels of SLC6A14 in plasma membrane led to increased leucine transport. These results show that the inactivation of AKT can rescue amino acid delivery through SLC6A14 trafficking to the cell surface, supporting cancer cell survival. The regulation of the ER export of the amino acid transporter seems to be a novel function of AKT.

## 1. Introduction

Quickly growing and proliferating cancer cells rely on the delivery of nutrients for anabolic processes and energy delivery [1]. Several solute carriers (SLCs) responsible for the uptake of such nutrients are upregulated in cancer. Beside the GLUT1–SLC2A1 transporting glucose, the main energetic substrate for aerobic glycolysis, there are several amino acid transporters upregulated in cancer, supporting not only protein synthesis, but also the biosynthesis of nucleotides and energy metabolism (for review, see [2]). Among these transporters are amino acid exchangers, such as SLC7A5 (LAT1) or SLC1A5 (ASCT2), whose activity deprives the cell of another amino acid [3]. One of the amino acids that is important for the reprogrammed metabolism of cancer cells is glutamine, which after glutaminolysis and the conversion to glutamate contributes to the production of several metabolites, as well as ATP and NADPH, which are all essential for anabolic processes (for review, see [4,5]). The glutamine transporters SLC38A1 (System A) and SLC38A5 (System N) are also upregulated in tumor cells. Glutamine can also be transported by SLC6A14—a transporter catalyzing net amino acid uptake, which leads to an increased amino acid concentration in cancer cells (for review see, [6]).

Solute carrier family 6 member 14 (SLC6A14) is also one of the amino acid transporters, it is called B^0,+^ (ATB^0,+^)—due to its broad substrate specificity, since it transports all neutral (index “0”) and basic (index “+”) amino acids. It belongs to the SLC6 family, comprising other amino acid transporters as well as transporters for monoamine neurotransmitters, γ-aminobutyric acid, creatine and osmolytes (for review, see [7]). SLC6A14 also transports some d-amino acids [8] and l-carnitine [9], a compound necessary for the transfer of acyl moieties to mitochondria for fatty acid β-oxidation [10]. SLC6A14 transports its substrates to the cell via symport with 2Na^+^ and 1Cl^−^, using transmembrane potential as well [11]. Originally, it was found to be highly expressed in the lungs, trachea and salivary glands [11]; later on, however, it was reported to be present in the intestinal tract [8,12], and in particular to be upregulated in solid tumors [13,14,15,16]. SLC6A14 has the highest affinity for the nonpolar amino acids isoleucine, leucine, methionine, valine and serine; however, glutamine and arginine are among its substrates [11], and it is worth mentioning that glutamine, arginine and leucine are especially important for the growth of cancer cells (for review, see [17]).

As with all transmembrane proteins targeting the plasma membrane, SLC6A14 is co-translationally inserted into the membrane of the endoplasmic reticulum (ER), and it undergoes quality control via chaperones. The ER-residing lectin calnexin controls protein conformation and the core glycosylation status [18]. The cytosolic part of SLC6A14 is controlled by a relay of heat shock proteins (HSPs): HSP70 (HSPA14) and HSP90-beta [19]. In order to reach the cell surface, a plasma membrane protein has to be exported from the ER and go through the *cis*- and *trans*-Golgi in the vesicles, which fuse with the plasma membrane. The initial step is the formation of membrane buds at so-called ER-exit sites, followed by the formation of vesicles coated with coatomer II (COPII), which, after scission from the ER, reach the endoplasmic reticulum-Golgi intermediate compartment (ERGIC) in metazoan cells. The budding of vesicles, as well as the formation of large membrane carriers for the transport of large-size cargo (procollagens), involves several proteins, and is started by Sec12, which converts Sar1 into its GTP-bound form, and this potentiates membrane curvature. This recruits Sec23/Sec24 heterodimers, and vesicle formation is completed after the binding of Sec13/Sec31 heterodimers that form heterotetramers as the outer lattice of the coat. The formation of these membrane carriers is coordinated by other proteins, such as Sec16 and Tango1/cTage5 complexes (for reviews, see [20,21,22,23]). The cargo protein, after quality control, interacts with the SEC24 proteins, of which there are four in human cells, named SEC24A-D. Out of these isomers, only SEC24C was shown to interact with SLC6A14 [24].

There is much information on the structure and interacting domains of COPII elements [21]. Much less is known, however, on the regulation of COPII formation. Posttranslational modifications, particularly *O*-glycosylation and phosphorylation, have been postulated to play a regulatory role [22]. Experiments with the recombinant and overexpressed SEC24C demonstrated that it was phosphorylated by serine/threonine protein kinase AKT (kinase B) [25]. It has to be noted that AKT is hyper-activated in many types of cancer, and it can suppress apoptosis, induce cell proliferation, inhibit tumor suppression, and activate glucose and lipid metabolism; moreover, AKT promotes tumor angiogenesis [26,27,28]. As shown by Sharpe et al. [25], the SEC24C phosphorylated by AKT co-precipitates with another COPII subunit, SEC23, and so AKT was proposed to regulate the trafficking and export from the ER [25]. Therefore, in the present study, we wanted to verify whether AKT can control the ER exit of SLC6A14 and its surface presence. The experiments were performed with cell lines characterized by a high level of SLC6A14, i.e., the endogenous transporter in breast cancer MCF-7 cells, and the overexpressed transporter in HEK293 cells.

## 2. Materials and Methods

### 2.1. Reagents and Materials

The l-[3,4,5,-^3^H(N)]Leucine (cat. no. NET460001MC) was from Perkin Elmer (Kraków, Poland). The rabbit Sec24C antibody (cat. no. 8531S) used for Western blots, as well as the rabbit polyclonal Akt antibody (cat. no. 9272) and the rabbit polyclonal phospho-Akt (Ser473) antibody (cat. no. 9271), were from Cell Signaling Technology (LAB-JOT LTD, Warsaw, Poland). The rabbit polyclonal SEC24C antibody (cat. no. NBP1-81550) for immunofluorescence was from Novus Biologicals (BIO-TECHNE, Warsaw, Poland). The biotin rabbit anti-SLC6A14 antibody (cat. no. 041972-Biotin) was from USBiological Life Sciences (VWR International, Gdańsk, Poland). The rabbit anti-SLC6A14 antibody for used Western blot was from Abcam (cat. no. ab99102, Symbios sp. z o. o., Straszyn, Poland). The streptavidin, Alexa Fluor™ 555 conjugate (cat. no. S-32355), goat anti-mouse IgG (H+L) highly cross-adsorbed antibody, Alexa Fluor™ 488 (cat. no. A-11029), goat anti-rabbit IgG (H + L) highly cross-adsorbed antibody, Alexa Fluor™ 568 (cat. no. A-11036), EZ-Link^®^ Sulfo-NHS-LC-Biotin (Sulfosuccinimidyl-6-(biotinamido)hexanoate) (cat. no. 21335), Pierce^®^ Avidin Agarose Resin (cat. no. 20225) and Roche phosphatase inhibitor Cocktail PhosphoSTOP™ (cat. no. 4906845001) were from Thermo Fisher Scientific (Life Technologies Polska, Warsaw, Poland), while the jetPRIME^®^ transfection reagent (cat. no. 114-07) was from Polypus Transfection (VWR International, Gdańsk, Poland). The MK-2206 (hydrochloride) (cat. no. 11593) was from Cayman Chemicals (BIOKOM, Warsaw, Poland). The monoclonal Anti-FLAG^®^ M2 antibody produced in mouse (cat. no. F3165), anti-FLAG antibody produced in rabbit (cat. no. F7425), monoclonal mouse anti-phosphoserine clone PSR-45 antibody (cat. no. P5747), monoclonal mouse anti-phosphothreonine antibody PTR-8 (cat. no. P6623), monoclonal anti-β-actin-peroxidase antibody (cat. no. A3854), IGEPAL CA-630 (Octylphenoxy poly(ethyleneoxy)ethanol, branched, cat. no. I8896), Duolink in situ detection kit (cat. no. DUO94104), protease inhibitor cocktail for use with mammalian cell and tissue extracts (cat. no. P8340), SC79 (cat. no. SML0749), and all other reagents were from Sigma (Poznań, Poland).

### 2.2. Cell Culture and Treatment

The HEK293 cells (cat. no. CRL-1573) purchased from ATCC^®^ (Mananas, VA, USA) were cultured in 10% fetal bovine serum and 90% Dulbecco’s modified Eagle medium, supplemented with penicillin (100 U/mL), streptomycin (100 µg/mL) and fungizone (0.25 µg/mL). Epithelial adenocarcinoma MCF-7 cells from ATCC^®^ were kindly provided by prof. Bożena Kamińska-Kaczmarek, Nencki Institute of Experimental Biology, Warsaw, Poland. The cell line was authenticated by ATCC^®^ in December 2019. The MCF-7 cells were cultured in 10% fetal bovine serum and 90% Eagle’s minimum essential medium supplemented with penicillin (100 U/mL), streptomycin (100 µg/mL) and fungizone (0.25 µg/mL). The cells were cultured at 37 °C in a humid atmosphere of 5% CO_2_.

The transfection of HEK293 cells with p3xFLAG-CMV14/B^0,+^ vector [29] was performed with jetPRIME^®^, according to the supplier’s protocol. After 24 h, either dimethylsulfoxide (DMSO) (vehicle) or the compounds indicated in the figure legends were administered to the DMSO for the indicated durations. The DMSO concentration did not exceed 0.1%. Both HEK293 and MCF-7 cells were lysed in 150 mM NaCl, 10 mM EDTA, 1% IGEPAL CA-630 and 50 mM Tris (pH 7.4), supplemented with protease inhibitor cocktail and PhosphoSTOP. The lysates were either subjected to Western blot analysis, with detection carried out via the antibodies indicated in the figure legends, or analyzed via two-dimensional (2D) gel analysis. Where applicable, the densitometric analysis was performed using a gel analysis tool in Fiji [30].

### 2.3. Two-Dimensional (2D) Gel Electrophoresis

After the treatment indicated in the figure legends, the cells were lysed, as described above, and the extracts were precipitated with 25% trichloroacetic acid. Further treatment and isoelectrofocusing were carried out as described in [31]. The strips were subjected to gel electrophoresis, and Western blots were probed with the indicated antibodies.

### 2.4. Immunocytochemistry and Proximity ligation Assay (PLA)

The cells were washed three times with phosphate-buffered saline (PBS) and fixed with methanol precooled to −20 °C. The cells were kept at −20 °C for 15 min, followed by three consecutive washes with PBS at room temperature. The unspecific binding sites were blocked for 2 h at room temperature with 5% goat serum in PBS. The cells were treated for 1 h at 4 °C with the two primary antibodies indicated in the figure legends. For the detection of SLC6A14 with biotin rabbit anti-SLC6A14 antibody, the streptavidin and Alexa Fluor™ 555 conjugate was applied; the detection of anti-SEC24C was performed with goat anti-rabbit IgG conjugated with Alexa Fluor™ 568. For the detection of phosphorylated amino acids, the corresponding goat anti-mouse IgG conjugated with Alexa Fluor™ 488 was used. The cells were washed three times with PBS and the samples were mounted in ProLong^®^ Diamond Antifade Mountant with 2-(4-amidinophenyl)-1H -indole-6-carboxamidine (DAPI). The cells were examined with the confocal microscope Zeiss LSM 800 (using 63× oil immersion objective), with an excitation of 493 nm and emission of 517 nm for the Alexa Fluor 488, an excitation of 561 and an emission of 603 nm for the Alexa Fluor 568, and an excitation of 553 nm and emission of 568 nm for the Alexa Fluor 555. An excitation of 353 nm and emission of 465 nm were used for the detection of DAPI.

PLA was used in order to verify the phosphorylation of the studied proteins. Fixed cells were incubated with the two primary antibodies indicated in the figure legends—one against the studied protein, the other against amino acid phosphorylated on either serine or threonine. The next steps were as follows: washing, incubation with assay probes, ligation with the ligase and amplification with polymerase, as per the supplier’s protocol. The analysis, after mounting in a medium with DAPI, was performed with a Zeiss LSM 800 spectral confocal microscope (using 40× oil immersion objective), with excitation set at 353 nm and emission at 465 nm for DAPI (nuclei) and excitation set at 561 nm and emission at 618 nm for Reagents Red. For endogenous proteins, the analysis was performed for at least 15 images for each coverslip, and the results were quantified and expressed as the red fluorescence signal per cell (nuclei number).

### 2.5. Biotinylation of Surface Proteins

After the experimental treatment described in the figure legend, the cells were washed with ice-cold PBS containing 0.1 M CaCl_2_ and 1 mM MgCl_2_ and subjected to a biotinylation procedure with the membrane impermeable reagent EZ-Link^®^ Sulfo-NHS-LC-Biotin (Sulfosuccinimidyl-6-(biotinamido)hexanoate) and the separation of biotinylated proteins with Pierce^®^ Avidin Agarose Resin, as described in [24].

### 2.6. Transport Measurements

The culture medium was removed and the cell monolayer was washed twice with the medium used for further experiment. PBS supplemented with 0.1 M CaCl_2_ and 1 mM MgCl_2_ was used for the control measurements, while 138 mM sodium gluconate, 2.7 mM potassium gluconate, 0.1 mM calcium gluconate and 1 mM magnesium gluconate were used in experiments without chloride. The cells were covered with the corresponding buffer and the transport of 90 nM [^3^H]leucine (5.7 Ci/mmol) was measured, as described in [32]. For the estimation of leucine transport, the cells were dissolved by incubating them overnight in 0.1 M NaOH, 2% Na_2_CO_3_ and 1% SDS, and samples were taken for radioactivity counting and protein estimation.

### 2.7. Statistical Analysis

Where applicable, the data are presented as mean ± standard deviation (SD). To test the significance of the differences between the control and experimental conditions, an unpaired *t*-test with Welch’s correction was applied, using GraphPad Prism version 6.07 software (GraphPad Software Inc., San Diego, CA, USA), with significance set at *p* < 0.05.

## 3. Results

### 3.1. The Inhibition of AKT Augments the Level of Slc6a14 in Plasma Membrane in HEK293 Cells

**Figure 1:**
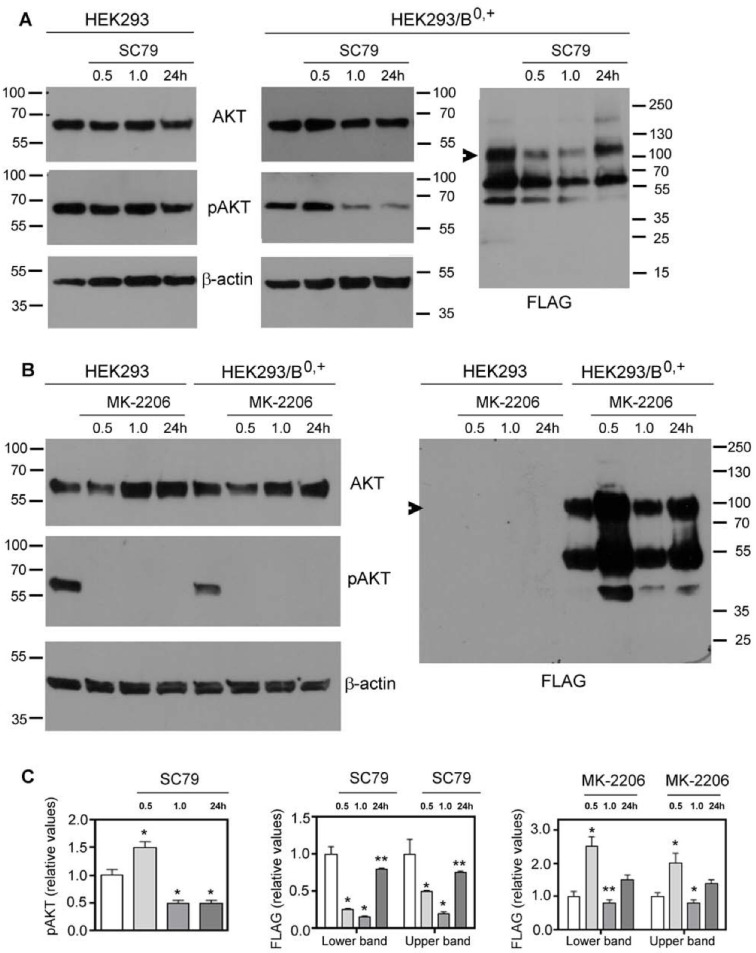
Effect of AKT on overexpressed SLC6A14. HEK293 cells or HEK293 cells transfected for 24 h with p3xFLAG-CMV14/B^0,+^ vector were treated for the indicated time with either 5 µg/mL SC79 (AKT activator) (**A**) or 5 µM MK-2206 (AKT inhibitor) (**B**). The blots were probed with the corresponding antibodies for the presence of AKT, AKT phosphorylated at Ser473 (pAKT), β-actin, and SLC6A14 (anti-FLAG). The representative blots out of 4 (**A**) or 3 (**B**) independent cell cultures are shown. Arrows indicate the fully glycosylated SLC6A14. (**C**) The integration of the indicated blots from (**A**,**B**) after transfection with p3xFLAG-CMV14/B^0,+^ vector. Significance of the respective controls of the lower and upper bands: * *p* < 0.01, ** *p* < 0.05.

The activation of AKT kinase starts with its phosphorylation on Thr308 via phosphoinositide-dependent kinase 1 (PDK-1) [33], followed by phosphorylation on Ser473 with a major contribution from the Rictor–mammalian target of rapamycin (mTOR) complex [34,35]. In order to check if AKT affects the surface presence of SLC6A14, we used the known AKT activator SC79 [36] and its inhibitor MK-2206 [37,38]. The level of active AKT was probed with anti-phosphoAKT antibodies. The treatment of HEK293 cells with SC79 for various durations did not change the level of phosphoAKT (Figure 1A, left panel), even at higher dose of 8 µg/mL (not shown). We even observed a 30–40% decrease in phosphoAKT after the longer treatment (24 h), resulting from a decrease in total AKT. These observations indicate that AKT is active under the applied experimental conditions. The transfection of HEK293 cells with a p3xFLAG-CMV14/B^0,+^ vector resulted, however, in a 40–50% increase in phosphoAKT after a 30 min treatment with SC79, while the longer incubation with the activator surprisingly reversed this effect (Figure 1A, middle panels). As shown previously in surface biotinylation and deglycosylation experiments [24], the analysis of SLC6A14 via Western blot indicates the localization of the transporter in the cell. The band of M_r_ 55,000–60,000 represents the non-glycosylated species residing in the ER, while the band of low-electrophoretic mobility represents the fully glycosylated transporter in the plasma membrane. Under the conditions at which phosphoAKT increased (0.5 h), we observed a lower amount of SLC6A14 migrating with low electrophoretic mobility (Figure 1A, right panel), a phenomenon slowly reversed when the level of phosphoAKT decreased, resulting in a level that was 80% of the control for both bands after the 24 h treatment with SC79. The treatment of HEK293 cells, either non-transfected or transfected with the p3xFLAG-CMV14/B^0,+^ vector, with AKT inhibitor MK-2206 was very effective, since for all tested durations the phosphoAKT could not be detected (Figure 1B, left panels). This confirmed the results obtained after SC79 treatment, indicating that AKT is already activated in HEK293 cells. It is interesting that MK-2206 treatment for 30 min resulted in a 20–30% decrease in total AKT, while longer treatment resulted in a 50 to 100% higher level of AKT when compared with the corresponding control. A lack of active AKT correlated with a two-fold increase in both SLC6A14 species after 30 min of treatment with MK-2206, although the amount of the transporter almost returned to the control level after 1 h of treatment, and the level of both bands increased by 40% after 24 h (Figure 1B, right panel, Figure 1C right panel). These observations indicate that the activity of AKT affects the level of SLC6A14 over short durations, most probably at the initial step of exit from the ER.

### 3.2. AKT Inactivation Does Not Protect SLC6A14 from Proteasomal Degradation

**Figure 2:**
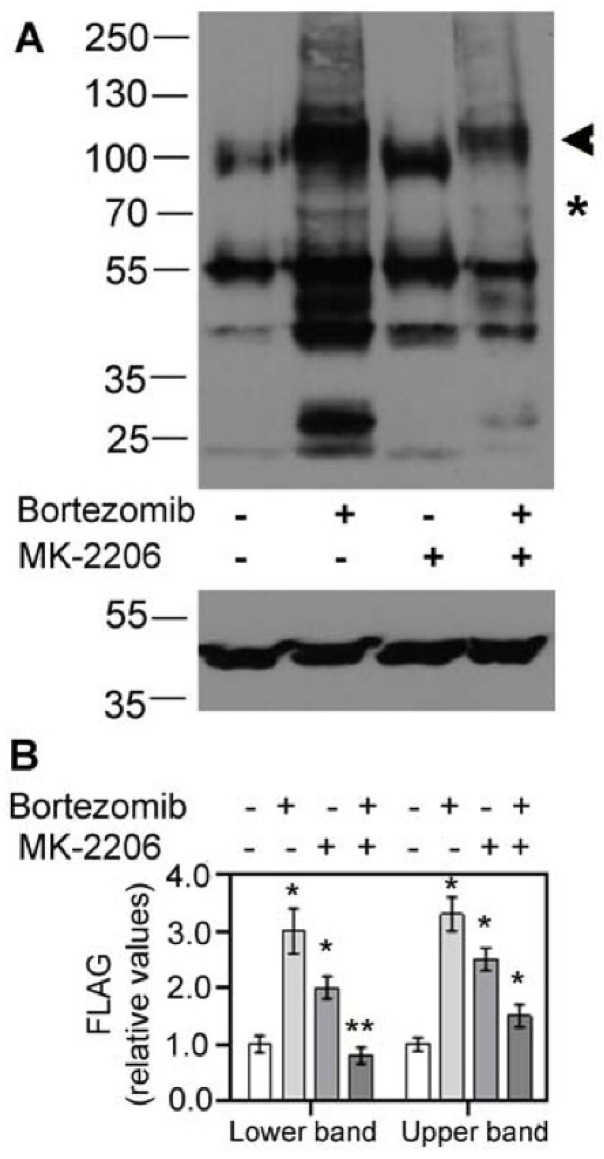
Effect of proteasomal degradation on SLC6A14. HEK293 cells were transfected for 24 h with p3xFLAG-CMV14/B^0,+^. Where indicated with “+” sign, 50 nM bortezomib was given for 24 h, while 5μM MK-2206 was added for the last 30 min, “−” means lack of the specified compound. (**A**) The upper panel shows the detection of SLC6A14 with anti-FLAG antibodies, while lower panel shows the detection of β-actin. The arrow indicates the fully glycosylated protein, the asterisk shows the core glycosylated species. (**B**) Integration of blots with FLAG detection. The analysis was performed with cells from 2 independent passages, and repeated for 2 different lysates from each passage. Significance of the respective control of the lower and upper bands: * *p* < 0.01, ** *p* < 0.05.

The dramatic increase in SLC6A14 upon AKT inhibition could be a result of the inhibition of ER-associated degradation (ERAD). Therefore, the cells expressing SLC6A14 were preincubated with the proteasome inhibitor bortezomib. As shown in Figure 2 and observed previously [24], a substantial amount of the overexpressed transporter is directed to ERAD, since we observe a 3-fold increase in bothSLC6A14 bands after bortezomib application (Figure 2B). On the contrary, although we observed an increase in high-molecular-weight SLC6A14 species upon AKT inhibition with MK-2206, the presence of bortezomib did not result in a further increase in the transporter level. Moreover, a 40–50% reduction in both bands could be observed when compared to MK-2206 treatment alone. This means that AKT does not influence the directing of SLC6A14 towards ERAD.

### 3.3. AKT Phosphorylates SLC6A14 and SEC24C

**Figure 3:**
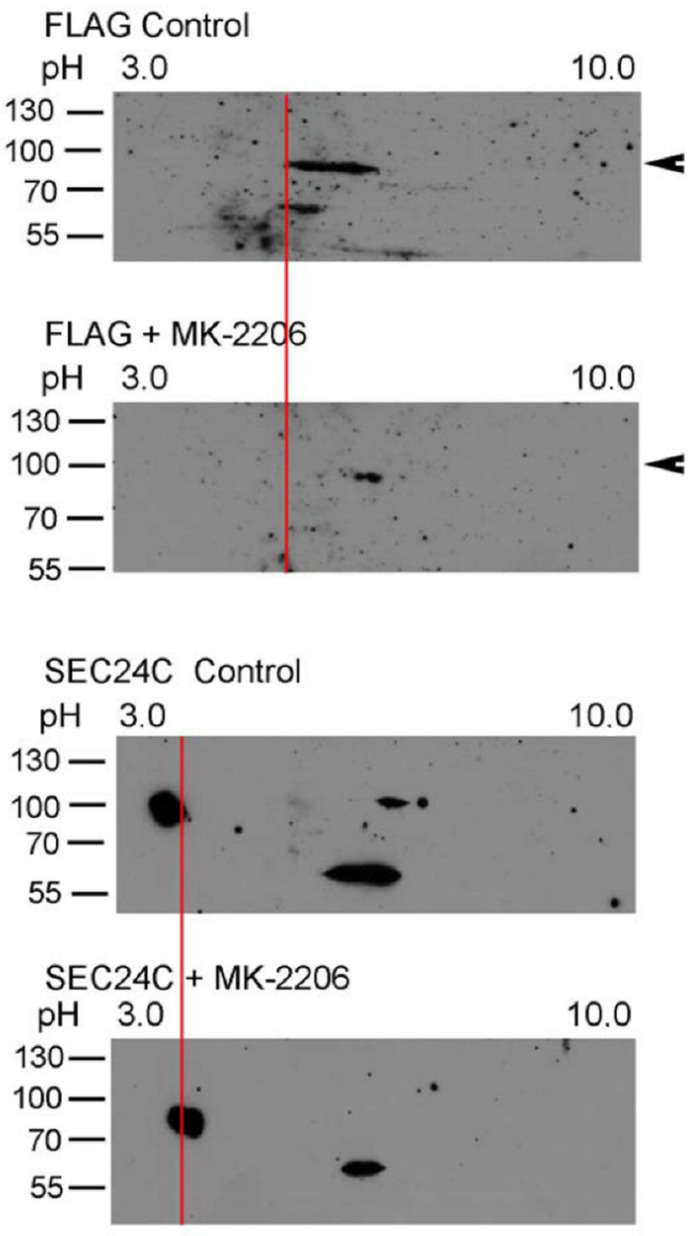
Two-dimensional analysis of SLC6A14 and SEC24C phosphorylation. HEK293 cells transfected for 24 h with p3xFLAG-CMV14/B^0,+^ were treated, where indicated, for 30 min with 5 μM MK-2206. The cells were lysed, as described in Section 2.2, and the lysates were analyzed via 2D electrophoresis. The change in pI is marked by a red line. The arrows show the migration of a fully glycosylated SLC6A14. Representative blots out of 4 independent experiments are shown.

AKT was shown to phosphorylate SEC24C [25]; therefore, we wanted to check whether AKT phosphorylates SLC6A14 and SEC24C under the applied experimental conditions. We applied 2D electrophoresis and, as presented in Figure 3, SLC6A14 was detected, as represented by the several spots. Treatment with MK-2260 shifted the upper band towards a more basicpI, which could suggest a reduction in phosphorylation or dephosphorylation. An analogous analysis of SEC24C also resulted in a shift of the upper spot to a more alkaline pI.

**Figure 4:**
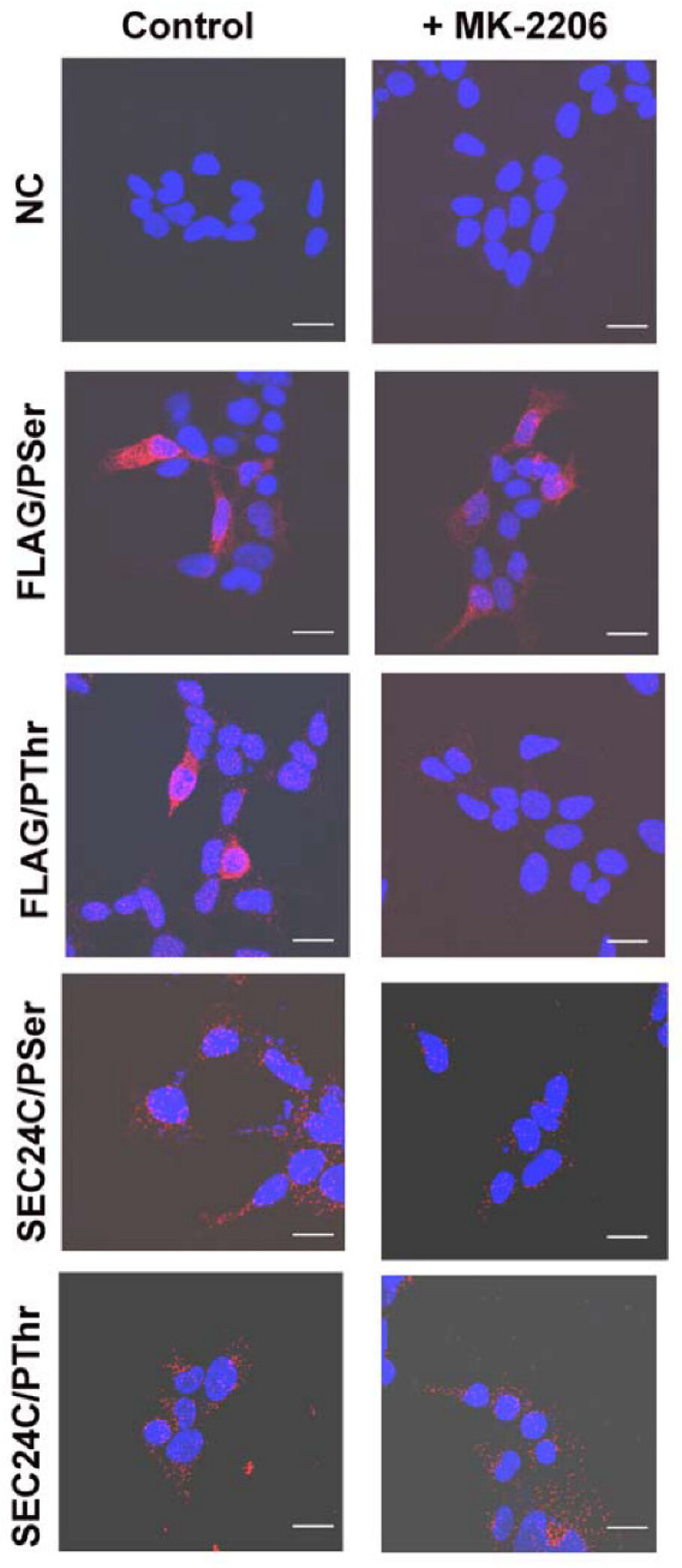
PLA analysis detecting phosphorylation with serine and threonine moieties on SEC24C and overexpressed SLC6A14. HEK293 cells transfected for 24 h with p3xFLAG-CMV14/B^0,+^ were treated, where indicated, for 30 min with 5 μM MK-2206, then fixed and subjected to PLA analysis. The following primary antibodies were used: rabbit anti-FLAG (1:1000) with either mouse anti-phosphoserine (1:250) or mouse anti-phosphothreonine (1:200), and rabbit anti-SEC24C (1:250) with either mouse anti-phosphoserine (1:250) or mouse anti-phosphothreonine (1:200), as indicated on the left. The presence of phosphorylated amino acid in the studied protein is visualized by the red dots, while the nuclei are shown in blue. NC, negative control; PLA, without the primary antibodies. The experiment was performed with 2 independent cell passages subcultured in 3 different coverslips each. The selected images come from the same passage. Bar: 20 μm.

We further used anti-phosphorylated amino acid antibodies to determine whether AKT phosphorylated serine and/or threonine moiety. The PLA assay demonstrated that SLC6A14 is phosphorylated on serine and on threonine under control conditions (Figure 4). However, MK-2206 treatment only reduced the signal in cases of anti-phosphothreonine antibodies, without affecting the signal detected with anti-phosphoserine antibodies, which would suggest that AKT phosphorylates SLC6A14 on threonine. We also detected the phosphorylation of SEC24C with serine and threonine (Figure 4). Regardless, the inhibition of AKT with MK-2206 resulted in a diminution of the phosphoserine signal (Figure 4).

### 3.4. AKT Controls Endogenous SLC6A14

**Figure 5:**
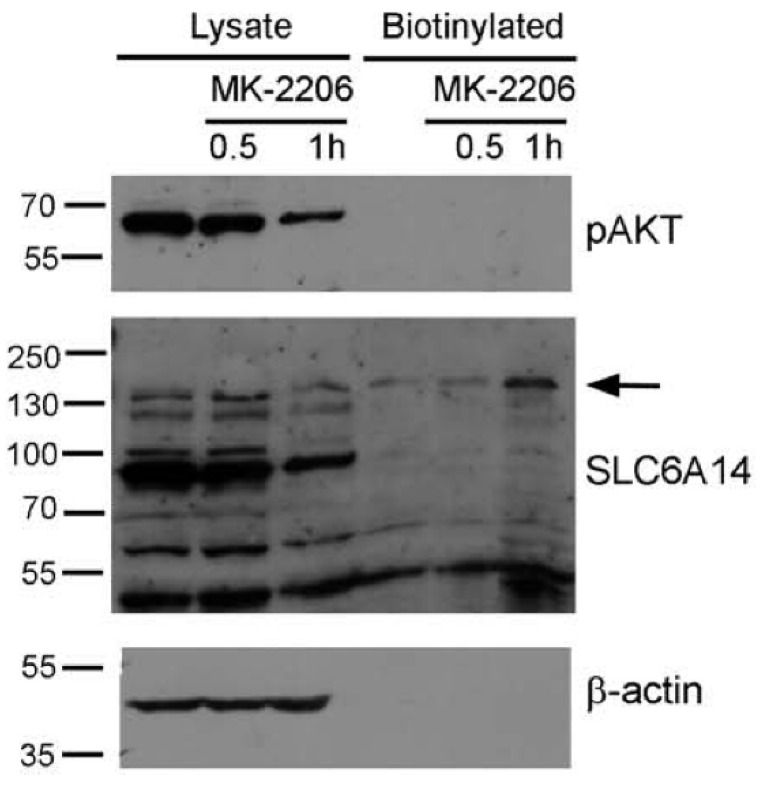
Effect of AKT inhibition on endogenous SLC6A14. MCF-7 cells were treated, where indicated, with 5 µM MK-2206. The cells were subjected to the biotinylation of surface proteins, as described in Section 2.5. The biotinylated fraction (from 800 µg protein) and the lysates (30 µg protein) were subjected to Western blot analysis and probed with anti-phosphoAKT antibodies (pAKT) and antibodies directed against SLC6A14 and β-actin. The representative blot out of 3 independent experiments is shown. The arrow indicates the presence of SLC6A14 in the plasma membrane.

The overexpressed plasma membrane transporter is directed to the cell surface, and so it highly depends on the regulation of ER export. We wanted to verify whether a similar level of involvement of AKT can be observed in the case of the endogenous transporter. Therefore, the estrogen receptor-positive breast cancer cell line MCF-7, known to express SLC6A14 [13], was selected. We observed previously [24] that the majority of SLC6A14 resides inside the MCF-7 cells. This observation was confirmed in the surface biotinylation experiment (Figure 5). An analysis of phosphorylated AKT shows that AKT was active in the MCF-7 cells, and a substantial (60%) decrease in phosphoAKT was detected after treatment with MK-2206 for 1 h (Figure 5, upper panel). This decrease in phosphoAKT is accompanied by the appearance of a biotinylated SLC6A14 band of low electrophoretic mobility, and the total SLC6A14 was lower after this treatment (Figure 5, middle panel). This indicates that the inhibition of AKT promotes the trafficking of the transporter to the cell surface.

**Figure 6:**
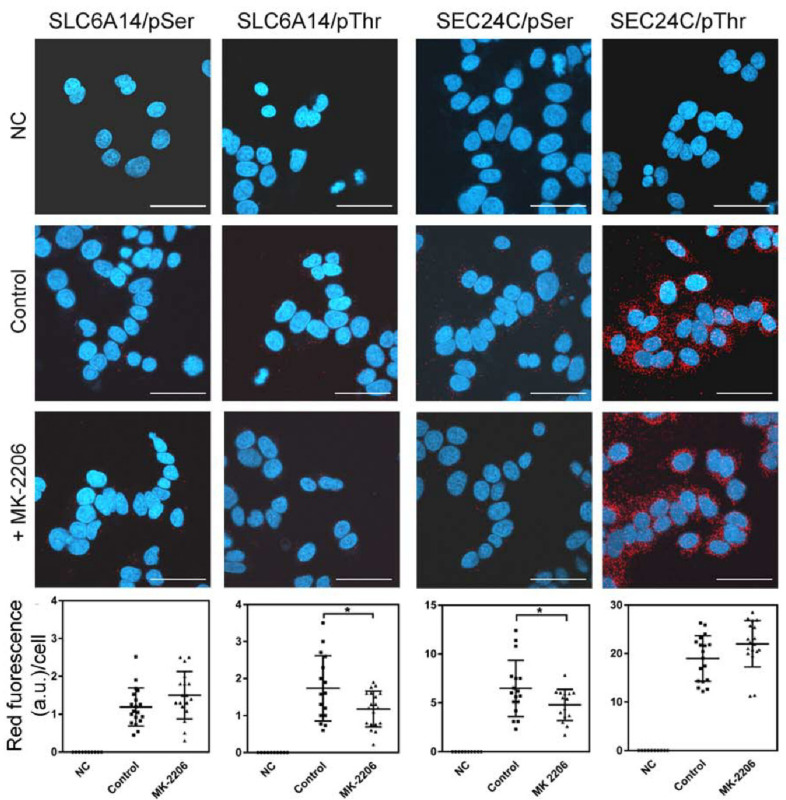
PLA analysis detecting phosphorylation on the serine and threonine moieties of endogenous SLC6A14 and SEC24C in breast cancer cells. MCF-7 cells were treated, where indicated, for 1 h with 5 μM MK-2206, then fixed and subjected to PLA analysis. The following primary antibodies were used: biotin rabbit anti-SLC6A14 antibody (1:250) with either mouse anti-phosphoserine (1:250) or mouse anti-phosphothreonine (1:200), and rabbit anti-SEC24C (1:250) with either mouse anti-phosphoserine (1:250) or mouse anti-phosphothreonine (1:200), as indicated. The presence of phosphorylated amino acid in the studied protein is visualized via the red dots, while nuclei are shown in blue. NC, negative control; PLA, without the primary antibodies. The representative images out of 2 independent cell cultures subcultured in 3 different coverslips each are shown. The selected images come from the same passage. Bar: 40 μm. The summarized results of the PLA assays are shown below. Statistical significance compared to the control at * *p* < 0.05.

The phosphorylated amino acids in the transiently overexpressed transporter could not be quantified; therefore, we verified the phosphorylation of serine and threonine moieties in endogenous SLC6A14 and SEC24C using the PLA assay. As shown in Figure 6, the phosphorylation of both amino acids was apparent in SLC6A14, although the detected signal, expressed in fluorescence arbitrary units (a.u.), was low (note the various ranges in Y axes) in comparison with the phosphorylation of SEC24C. This difference may reflect the various levels of both proteins in MCF-7 cells. Under control conditions, the phosphoserine signal in SLC6A14 was 1.19 ± 0.12 a.u./cell, while for MK-2206 it was 1.50 ± 0.15 a.u./cell; however, this difference was not statistically significant. The SLC6A14 phosphorylation of threonine was higher under control conditions (fluorescence 1.74 ± 0.22 a.u./cell) and significantly decreased after MK-2206 treatment (fluorescence 1.18 ± 0.11 a.u./cell). In the case of SEC24C, we detected a massive signal of phosphothreonine; however, this did not change significantly between control conditions and the treatment with the AKT inhibitor (fluorescence 19.0 ± 1.1 a.u./cell vs. 22.0 ± 1.1 a.u./cell, respectively). Although the level of SEC24C phosphorylated on the serine moiety was lower than that phosphorylated on threonine, incubation with MK-2206 reduced this signal significantly from 6.48 ± 0.70 a.u./cell to 4.78 ± 0.40 a.u./cell. These results confirm observations of the phosphorylation of both proteins after SLC6A14 overexpression.

**Figure 7:**
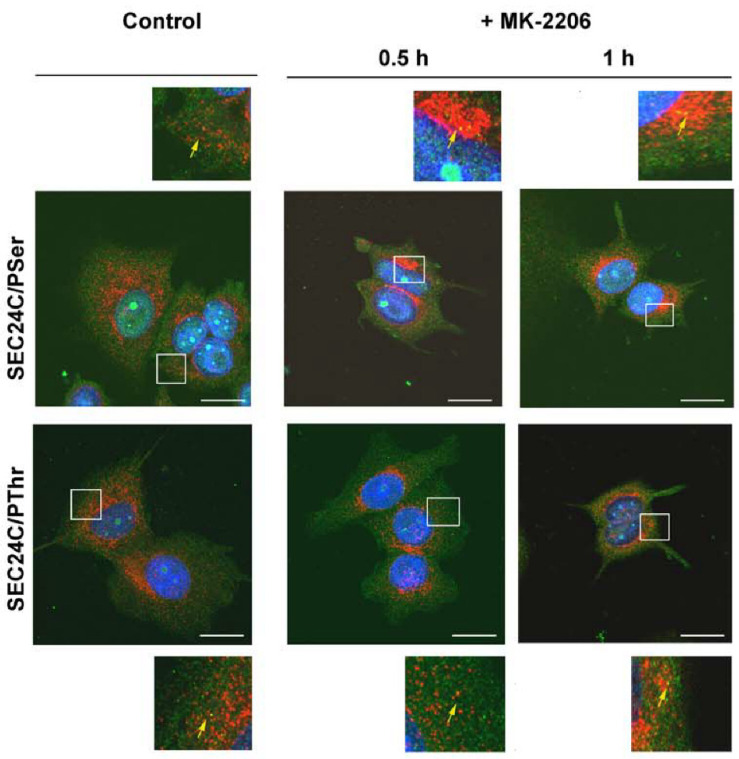
Immunocytochemistry analysis of SEC24C phosphorylation in MCF-7 cells. Untreated cells or cells incubated for the indicated time with 5 μM MK-2206 were fixed and subjected to the immunocytochemistry procedure, as described in Section 2.4. The following primary antibodies were used: rabbit anti-SEC24C (1:250, red) with either mouse anti-phosphoserine (1:250, green) or mouse anti-phosphothreonine (1:200, green), as indicated on the left. The selected areas are shown as magnified images either above or below the corresponding panels. The detected co-localizations of SEC24C and phosphorylated amino acid are indicated with yellow arrows. Representative images from 2 independent cell passages subcultured in 3 different coverslips each are shown. The selected images come from the same passage. Bar: 20 μm.

**Figure 8:**
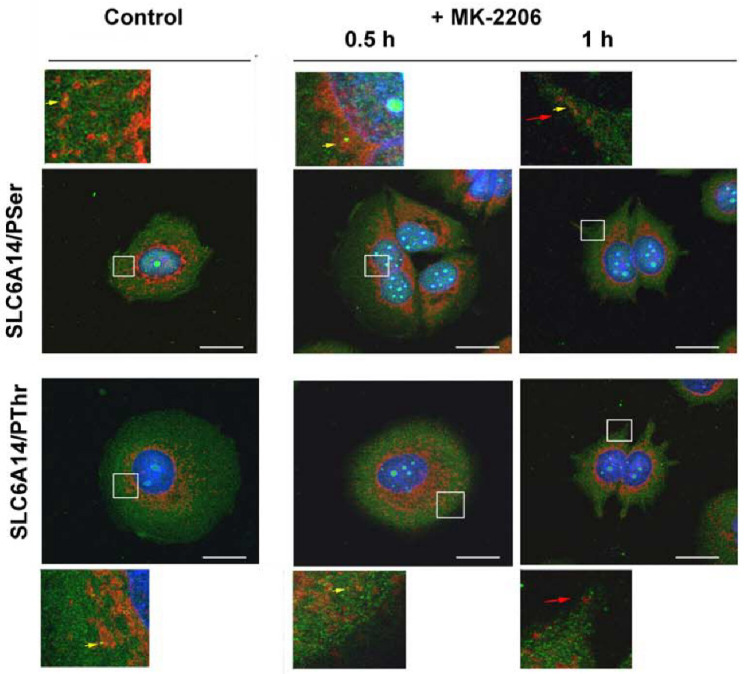
Immunocytochemistry analysis of SLC6A14 phosphorylation in MCF-7 cells. Untreated cells or cells incubated for the indicated time with 5 μM MK-2206 were fixed and subjected to the immunocytochemistry procedure, as described in Section 2.4. The following primary antibodies were used: rabbit anti-SLC6A14 (1:250, red) with either mouse anti-phosphoserine (1:250, green) or mouse anti-phosphothreonine (1:200, green), as indicated on the left. The selected areas are shown as magnified images either above or below the corresponding panels. The detected co-localizations of transporter and phosphorylated amino acid are indicated with yellow arrows, and the surface presence of SLC6A14 with red arrows. Representative images from 2 independent cell passages subcultured in 3 different cover slips each are shown. The selected images come from the same passage. Bar: 20 μm.

We further analyzed the localization of SLC6A14 and SEC24C in MCF-7 cells upon AKT inhibition. As presented in Figure 7 and Figure 8, and as was easy to predict, the presence of the phosphorylated amino acids can be observed in the whole cells. SEC24C is spread out across the cell under control conditions (Figure 7), while under MK-2206 treatment it is concentrated close to the nuclei. Both SEC24C phosphorylated on serine and SEC24C phosphorylated on threonine could be detected; the phosphorylated forms were observed in vesicles in the controls, while after MK-2206 co-localization with phosphoserine, the signal was exclusively visible in regions in the vicinity of nucleus. SLC6A14 was mainly detected inside the cell, while its phosphorylated forms, on both the serine and threonine moiety, were visible on vesicles located more distally (Figure 8). The treatment of MCF-7 cells with MK-2206 resulted in the spreading-out of the transporter to the cell surface, with SLC6A14 detected in the plasma membrane, and mainly in protrusions, after 1 h (indicated by red arrows). The co-localization of the transporter with phosphoserine can also be detected in cell protrusions at the cell surface (Figure 8, upper far right panels, yellow arrow), while the signal of SLC6A14 phosphorylated on threonine was not visible after 1 h of treatment with MK-2206.

### 3.5. Inhibition of AKT Increases SLC6A14 Activity

**Figure 9:**
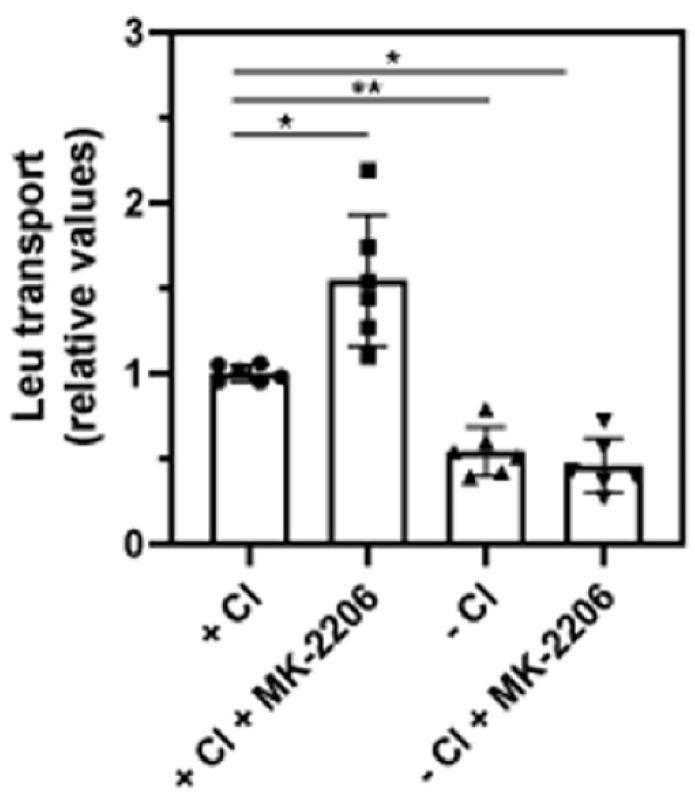
Leucine transport in MCF7 cells. Transport of [^3^H]leucine was measured for 10 min, as described in Section 3.5, either in PBS (+Cl) or in gluconate solution (−Cl), with or without 30 min treatment with MK-2206, where indicated. The results of 6 independent measurements with different cell cultures are shown. * *p* < 0.01, ** *p* < 0.05.

The appearance of the high-molecular-weight form of SLC6A14, as well as the results of the biotinylation experiment and immunocytochemistry analysis, indicate an augmented quantity of transporter in the plasma membrane when AKT was inhibited. Therefore, we wanted to verify whether this correlated with increased transport activity. Leucine was chosen, due to its high affinity to SLC6A14. This amino acid can also transported by other proteins, mainly by members of the SLC7 family. Human breast cancer cells, including MCF-7, were shown to express LAT1 (SLC7A5) and LAT2 (SLC7A8) [39]; however, only SLC6A14 requires chloride ions for its activity. Therefore, the transport of leucine was measured in the presence of Cl^-^ in PBS or in gluconates solution (Figure 9). The omission of Cl^-^ resulted in a significant reduction in transport activity to 54 ± 4% of the total leucine uptake in the presence of chloride. This indicates the substantial contribution of SLC6A14 to the uptake of leucine. MK-2206 treatment increased transport by 55 ± 11% in the presence of chloride; however, it did not affect other transporters when the uptake of leucine was measured without chloride.

## 4. Discussion

Our results show that both SLC6A14 and SEC24C are phosphorylated by AKT. AKT phosphorylates SLC6A14 on threonine. The observed phosphorylation on serine, which is not affected by AKT inhibition, corresponds to the previously detected phosphorylation by protein kinase C (PKC) [29]. Although an in silico analysis with the NetPhos 3.1 Server (http://www.cbs.dtu.dk, accessed on 28 January 2019) indicated Thr^759^ of SEC24C as a possible AKT target (score 0.717), we observed a decrease in phosphoserine signal upon AKT inhibition. Interestingly, it was proposed that SEC24C is phosphorylated in the C-terminal region after the truncation of the first 800 amino acids [25], which excludes Thr^759^.

It is interesting that the endogenous SLC6A14 phosphorylated on serine appears close to/at the cell surface upon AKT inhibition. We previously observed an augmented level of phosphoserine in the overexpressed SLC6A14 after the activation of PKC, and we detected the co-localization of PKCα with the transporter at the plasma membrane [29,32]. AKT and PKC require priming phosphorylation with PDK-1; moreover, both kinases are further phosphorylated by the mTORC2 complex [40]. mTORC2 phosphorylates all conventional PKCs, and phosphorylation on a turn motif of PKCα and Akt is dependent on the mTORC2 subunit Rictor [41]. The mutual relationship between PKC and AKT is more complicated—it depends on cell type and PKC isoform. The activation of PKC with phorbol ester reduced the phosphorylation of AKT in 11 out of 21 distinct cell lines [42]. It has also been shown that PKCδ and PKCε cause Akt dephosphorylation, while PKCα enhanced the Ser473 phosphorylation of Akt [43]. On the other hand, the effect of AKT inhibition on PKC activity remains obscure; we cannot exclude, however, that mTORC2 is more available for PKC when AKT is inactive. The intracellular localization of both kinases may also be important to mTORC2 availability, especially considering that active PKCα was detected in the plasma membrane [29], while the phosphorylation of SEC24C and SLC6A14 by AKT most probably takes place at the ER exit. Sharpe et al. [25] showed the co-precipitation of SEC24C with SEC23 upon activation of the AKT pathway, and a reduction in this interaction when AKT was inhibited. They proposed that such an interaction could regulate the trafficking of cargo. Via immunofluorescence imaging, we can see that SEC24C condensed close to the nuclei when AKT was inhibited, which probably reflects the localization of COPII-coated vesicles close to the ERGIC, a compartment known to be only few hundred of nanometers away from the ER [44]. The concomitant appearance of the transporter at the cell surface suggests the quick release of cargo after uncoating. The process of uncoating has been proposed previously to be initialized by the phosphorylation of Sec23 and Sec31 by casein kinases [45,46]. We cannot exclude the possibility that SEC24C responds more quickly to the process of uncoating at the ERGIC when phosphorylation of its serine moiety is diminished, which represents another control step in the trafficking of cargo to the cell surface.

AKT and the phosphatidyl-3-kinase/AKT/mTOR pathway are hyperactivated in various cancers by upstream regulatory proteins. This affects the downstream effectors that control cell growth, survival, proliferation, metabolism, angiogenesis and metastasis [28,47]. Apart from being activated by mTORC2 [48], AKT is also involved in the mTORC1 pathway—active AKT phosphorylates heterodimer tuberous sclerosis complex 1, which inhibits the activation of small GTPase Rheb and results in the activation of mTORC1, leading to the activation of the translation machinery [48,49]. It should be noted that mTORC1 is activated by amino acids from the lysosome (for review, see [50]), due to the activity of SLC38A9, a lysosomal amino acid transporter [51,52]. mTORC1 can also be regulated by extracellularly added amino acids [50,53]. In the case of AKT inhibition and the augmented surface presence and activity of SLC6A14, amino acids (including the leucine and arginine activating mTORC1) will be transported from outside the cell. AKT has also been shown to regulate endo- and exocytosis. In particular, after binding to phosphatidylinositol 3,4,5-trisphosphate, it was shown to increase the vesicular trafficking of glucose transporter GLUT4 to the plasma membrane via its action on Rab GTPase-activating proteins [54]. We observed the opposite effect, detecting more SLC16A14 in the plasma membrane when AKT was inhibited. This would point to AKT as causing the switch between glucose metabolism and increased amino acids uptake, depending on its activity status. This would create a protection mechanism for cell survival, which is especially important in the case of cancer cells. Interestingly, AKT is dephosphorylated by PP2A [55], a phosphatase whose active form has been shown to promote the trafficking of another SLC transporter to the cell surface, namely, SLC22A5 [31], a carnitine transporter the high level of which is correlated with the β-oxidation of fatty acids. Of note, active β-oxidation supports cancer cells in the anabolic processes delivering not only the energy (ATP synthesis) but also the NADH, NADPH and FADH_2_ required for quick cell growth and proliferation in cancer (for review, see [56]).

In conclusion, our observations show that through the phosphorylation of SEC24C and SLC6A14, AKT can regulate the trafficking of the transporter to the cell surface at the ER exit, which is a novel physiological function of this kinase. Moreover, since the activity of SLC6A14 does not deprive cells of other amino acids, as in the case of exchangers, this regulation is important for cell growth, a process crucial for cancer cells in which AKT is hyper-activated (for review, see [57]) [27]. It seems that, depending on the level of AKT activity, cancer cells rely on either glucose, as an energetic substrate, or amino acids and the β-oxidation of fatty acids for their survival. This would make AKT a central node in the switching of cancer cell metabolism by enabling adaptation to nutrient availability. One should bear this in mind, since AKT inhibitors have been tested in clinical trials for the treatment of tumors [58], and so combination therapies with both AKT and SLC6A14 inhibitors will presumably be more promising.

## Data Availability

Data is contained within the article.
